# Biomechanical Analysis of Woodpecker Response During Pecking Using a Two-Dimensional Computational Model

**DOI:** 10.3389/fbioe.2020.00810

**Published:** 2020-07-17

**Authors:** Shailesh Ganpule, Sunil Sutar, Kaustaubh Shinde

**Affiliations:** Department of Mechanical and Industrial Engineering, Indian Institute of Technology Roorkee, Roorkee, India

**Keywords:** woodpecker, human, pecking, impact biomechanics, brain injury, scaling

## Abstract

Traumatic brain injury (TBI) and chronic traumatic encephalopathy (CTE) due to the impact is a critical health concern. Impact mitigation strategy is a vital design paradigm to reduce the burden of TBI and CTE. In this regard, woodpecker biomimicry continues to attract attention. However, a direct comparison between a woodpecker and human biomechanical responses is lacking. Toward this end, we investigate the biomechanical response of a woodpecker during pecking using a two-dimensional head model. We also analyze the response of concurrent human head model to facilitate direct comparison with woodpecker response. The head models of woodpecker and human were built from medical images, the material properties were adopted from the literature. Both woodpecker and human head models were subjected to head kinematics obtained during pecking and resulting biomechanical response is studied. For the pecking cycle simulated in this work, peak rotational velocity and acceleration were ∼15 rad/s and 7,057 rad/s^2^. These peak values are commensurate with the kinematics threshold values reported in human TBI. Our results show that, for the same input acceleration, the strains and stresses in the woodpecker brain are approximately six times lower than that of the human brain. The stress reduction is mainly attributed to the smaller size of the woodpecker head. The effect of pecking frequency and multiple pecking cycles have also been studied. It is observed that the strains and stresses in the brain are increased by ∼100% as pecking frequency is doubled. During multiple pecking cycle, dwell period of ∼90 ms tend to relax the stresses in the woodpecker brain; however, the amount of relaxation depends on the value of the decay constant. The comparison of biomechanical response against the axonal injury threshold suggests that for peak rotational acceleration of 7,057 rad/s^2^ the maximum principal strain in the brains of woodpecker and human exceed the threshold limit. Acceleration scaling relationship between a woodpecker and equivalent human response is also developed as a function of head size. We obtain a scaling factor, $\frac{a_{\text{h}}}{a_{\text{w}}}$, of 0.11 for baseline head sizes and a scaling factor of 1.03 as the human head size approaches woodpecker head size.

## Introduction

The emergence of Traumatic brain injury (TBI) and Chronic traumatic encephalopathy (CTE) among American Football players and returning soldiers have created a sense of urgency toward the mitigation and prevention of these injuries (Meaney et al., 2014). In this regard, woodpecker continues to attract attention in terms of scientific curiosity (e.g., May et al., 1976; Gibson, 2006; Liu et al., 2017) and biomimicry (e.g., Lemire, 2017). From the biomechanics perspective, based on either theoretical or finite element analysis, several theories have been proposed on how woodpecker avoids brain injury. These theories are: (a) small size of the woodpecker brain (Gibson, 2006) (b) presence of long beak (Wang et al., 2011; Liu et al., 2017), (c) presence of hyoid bone (Wang et al., 2011; Zhu et al., 2012; Liu et al., 2015b), and (d) dome shaped skull (Zhu et al., 2012). Even though these investigations are encouraging, they do not take into account a few aspects, as identified below. (i) These investigations do not take into account the entire pecking cycle and consider response only when woodpecker impacts the tree. Thus, the role of full pecking cycle, including the role of rotational motion of the woodpecker’s head, on brain response is unknown. It is well established in TBI literature that the rotational motion plays a critical role in generating diffuse axonal injuries (Margulies et al., 1990; Wright et al., 2013; Ji et al., 2014; Ganpule et al., 2017). Note that several investigations (Vincent et al., 2007; Liu et al., 2015a) studied a full pecking cycle with a focus on understanding head kinematics. Present work evaluates the kinetics of the head (including brain) during a full pecking cycle. (ii) The total simulated time in most of the woodpecker biomechanics investigations is a few milliseconds; with such a small simulated time, stress wave propagation effects are not fully played out within the brain (Ganpule et al., 2017). (iii) These investigations lack direct one-to-one comparison with the human biomechanical response. (iv) Some of the investigations (Wang et al., 2011; Zhu et al., 2012) have focused on mechanisms protecting the woodpecker from injury with little to no focus on the injury and comparison of biomechanical response against existing injury thresholds. A recent investigation has found the accumulation of tau-protein in the heads of woodpeckers (Farah et al., 2018) as compared to the control (red-winged black bird). Accumulation of tau-protein is a neuropathology implicated in human CTE (McKee et al., 2016). Thus, the question of whether woodpecker avoids brain injury has re-emerged with serious implications in biomimicry (Smoliga, 2018; Smoliga and Wang, 2019).

The goal of this work is to study the biomechanical response of woodpecker during the pecking process with emphasis on addressing aforementioned gaps in the literature. We also seek to compare the biomechanical response of a woodpecker with a human under the same set of loading and boundary conditions. Toward this end, we have built two-dimensional finite element models of woodpecker and human. We perform detailed biomechanical analysis under the same set of loading and boundary conditions taking into account woodpecker’s pecking process.

## Materials and Methods

### Finite Element Discretization

**FIGURE 1:**
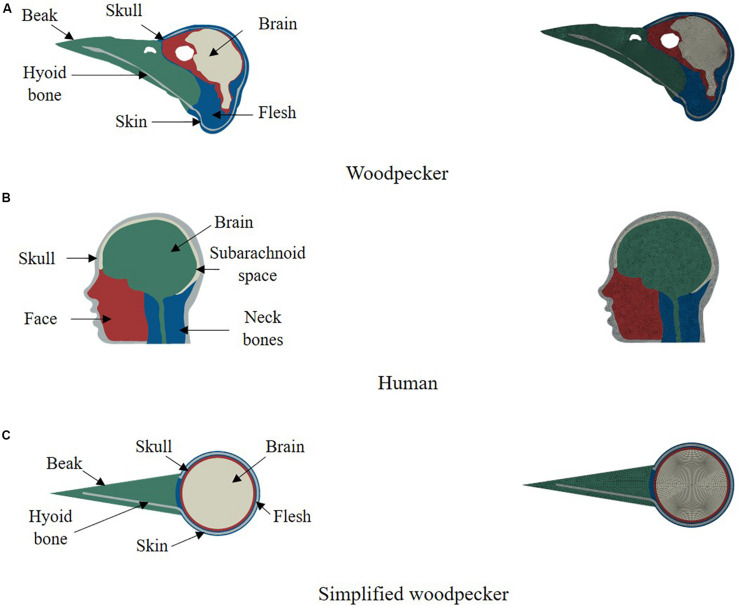
2D computational models of **(A)** woodpecker, **(B)** human, and **(C)** simplified woodpecker.

We have used finite element method to simulate biomechanical response of woodpecker and human. Finite element method has widely been used to simulate brain biomechanics and is well verified and validated (e.g., Yang et al., 2011; Mao et al., 2013; Giordano and Kleiven, 2014; Ji et al., 2015). Two dimensional (2D), plane strain, finite element model of a woodpecker head (Figure 1A) was built from the midsagittal CT image obtained from the Digital Morphology library at the University of Texas at Austin (DigiMorph, 2004). Based on the intensity of pixels and knowledge of woodpecker geometry, the image was segmented (Materialize Mimics^®^) into five regions namely skin (flesh), skull, beak, hyoid bone, and brain. 2D, plane strain, finite element head model of human head (Figure 1B) was built from the midsagittal MRI image obtained from Visible Human Project (National Institutes of Health, 2009). The human model was segmented (Materialize Mimics^®^) into five regions namely skull, facial tissue, neck bones, subarachnoid space, and brain. The threshold value of 300 HU was used to delineate the soft (skin, brain, and subarachnoid space) and hard (skull, beak, hyoid, and neck bones) tissues. The segmented models of woodpecker and human were meshed with an average mesh size of ∼0.2 mm, ∼2 mm, respectively, using HyperMesh^®^. This resulted in 19,084 and 9,280 elements for the woodpecker and human head models, respectively. At these mesh resolutions, the mesh is converged (<5% difference in peak stresses and strains), the results of mesh convergence are shown in Supplementary Figure S1. In addition, a simplified model of a woodpecker was built (Figure 1C) with mesh resolution of ∼0.2 mm to study the effects of geometrical features on brain response. For all models, four noded, plane strain, reduced integration elements (CPE4R) were used. Due to the complex geometry, there were a few (<5%) three noded, plane strain elements (CPE3) present in these models. For all models, the interface between various segmented regions was perfectly bonded (no tangential sliding, no separation).

### Material Models

Brain tissue was modeled as a hyperelastic solid using Ogden strain energy function, which has the following form.

(1) $$U=\sum_{i=1}^{N}\frac{2\,\mu_{\text{i}}}{\alpha_{\text{i}}^{2}}\,\left({\bar{\lambda }}_{1}^{\alpha_{\text{i}}}+{\bar{\lambda }}_{2}^{\alpha_{\text{i}}}+{\bar{\lambda }}_{3}^{\alpha_{\text{i}}}-3\right)$$

Where, μ_*i*_ are the shear moduli, α_i_ are the material constants (fitting parameters), and ${\bar{\lambda }}_{\text{i}}=\frac{\lambda_{\text{i}}}{\sqrt[{3}]{J}}$ are the deviatoric principal stretches. *J* and λ_*i*_are the Jacobian and principal stretches, respectively. Note that, for the material properties of the brain tissue used in this work *N* = 1. Time dependent behavior of brain tissue is modeled using quasilinear viscoelastic function.

(2) $$\mu \,\left(t\right)=\mu_{0}\times \left[1-\sum_{i=1}^{N}g_{i}\,\left(1-e^{-t/\tau_{\text{i}}}\right)\right]$$

Where, μ_*0*_ is the instantaneous shear modulus and *g*_*i*_, and τ_*i*_ are the material constants. Material properties for the woodpecker brain are not available in the literature. Thus, the material properties for the woodpecker brain used in this work are based on characterization in human and porcine brains, consistent with the other investigations in the literature (Wang et al., 2011; Zhu et al., 2012; Liu et al., 2015b, 2017). We have also studied the sensitivity of model response to various material properties of the brain tissue reported in the literature (Supplementary Table S1).

All other tissues of the head were modeled as elastic solid, consistent with the head biomechanics literature (Wang et al., 2011; Zhu et al., 2012; Mao et al., 2013; Giordano and Kleiven, 2014; Ji et al., 2015; Liu et al., 2015b, 2017; Ganpule et al., 2017). The material properties used in the head model are tabulated in Table 1.

**TABLE 1:** Material properties used in head model.

Substructure	Properties	Source
**(A) Woodpecker**		
Beak	$\rho =1456\,\frac{k\,g}{m^{3}}$; *E* = 1*GPa*; υ = 0.3	Liu et al., 2017
Hyoid bone	$\rho =1040\,\frac{k\,g}{m^{3}}$; *E* = 3.72*GPa*; υ = 0.4	Liu et al., 2017
Skull	$\rho =1456\,\frac{k\,g}{m^{3}}$; *E* = 0.31*GPa*; υ = 0.4	Liu et al., 2017
Flesh	$\rho =1070\,\frac{k\,g}{m^{3}}$; *E* = 1*MPa*; υ = 0.45	Liu et al., 2017
Brain	1st-order Ogden hyperelastic: ρ = 1040kg/m^3^ μ_0_ = 2780Pa, μ_∞_ = 303.3*Pa*, α = 6.0, *g*_1_ = 0.5663, *g*_2_ = 0.3246, τ_1_ = 0.0350,τ_2_ = 0.0351	Rashid et al., 2014
**(B) Human**		
Skull	$\rho =2070\,\frac{k\,g}{m^{3}}$; *E* = 8*GPa*; υ = 0.22	McElhaney et al., 1970
Face	$\rho =2000\,\frac{k\,g}{m^{3}}$; *E* = 15*GPa*; υ = 0.22	Kleiven and Hardy, 2002
Neck	$\rho =1300\,\frac{k\,g}{m^{3}}$; *E* = 1*GPa*; υ = 0.24	Kleiven and Hardy, 2002
Subarachnoid space	$\rho =1133\,\frac{k\,g}{m^{3}}$; *E* = 9.85*MPa*; υ = 0.49	Jin et al., 2006
Brain	1st-order Ogden hyperelastic: ρ = 1040kg/m^3^ μ_0_ = 2780Pa, μ_∞_ = 303.3*Pa*, α = 6.0, *g*_1_ = 0.5663,*g*_2_ = 0.3246, τ_1_ = 0.0350,τ_2_ = 0.0351	Rashid et al., 2014

### Pecking Cycle and Loading Conditions

**FIGURE 2:**
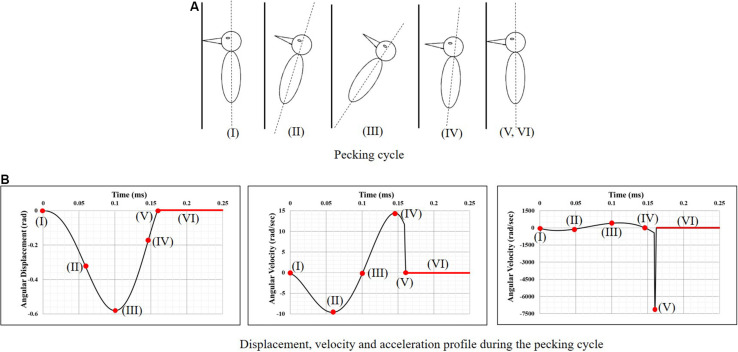
Woodpecker’s head kinematics during the pecking cycle. **(A)** Pecking cycle and **(B)** displacement, velocity and acceleration profile during the pecking cycle (Vincent et al., 2007).

Vincent et al. (2007), using video footage and spring-mass-damper based kinematic model of a woodpecker, obtained complete kinematics of woodpecker’s head and body during the pecking cycle. Broadly, the pecking cycle is divided into the following phases (Figure 2), based on the kinematics. (I) Initial contact with wood: woodpecker’s beak is still in contact with the tree following the past cycle. At this instant, the woodpecker is stationary and keeps up its vertical position by holding onto the tree with its legs which act as a gripper. The motion is initiated after this point. (II) Halfway rotation completed: the bird keeps on moving far from the tree, with the claws providing the necessary force. (III) Extreme end reached and motion is reverted: by this time, the woodpecker has reached the extreme end of its swing. The claws are forced by the muscles and the bird begins to move toward the tree. The underbody moves closer to the wood and the reaction load between tail quills and wood provides the extra rotational power to the body. (IV) Before an impact with the tree: maximum velocity is reached slightly before woodpecker impacts the tree providing the highest momentum. (V) Impact with the tree: the bird reaches its maximum deceleration at this instant and hits the wood. The gripper reduces its force of grabbing the tree bark so that the whole momentum is transferred to the tree via the beak. As there is a sudden stop in rotational motion at this point, the bird experiences a severe angular deceleration. (VI) Dwell period: once woodpecker impacts the tree, there is a dwell period of ∼90 ms before motion is reversed.

**FIGURE 3:**
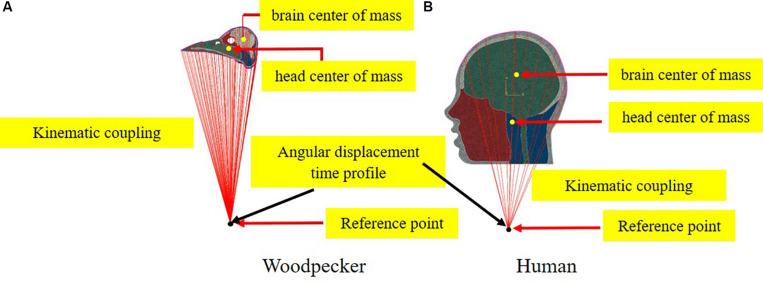
Schematic depicting the application of boundary conditions **(A)** woodpecker and **(B)** human.

To simulate the full pecking cycle, the angular displacement-time history (Figure 2B) obtained by Vincent et al. (2007) was applied to all the head models and the corresponding model response was studied. In the case of woodpecker, the angular displacement-time history was applied at the reference point (Figure 3A) located at a vertical distance of 117 mm from the center of mass of the head based on the video analysis of woodpecker’s pecking process (Vincent et al., 2007). In case of human, the angular displacement-time history was applied at a reference point (Figure 3B) located at the base of the neck (191 mm from the center of mass of the head). Head rotation studies in human volunteers suggest that head rotates about the neck (Sabet et al., 2008; Ganpule et al., 2017; Gomez et al., 2018). In all the models, the outer surface (edge in case of 2D) of the head model is kinematically coupled to the reference point as shown in Figure 3. The key kinematical parameters during each phase of the pecking cycle are tabulated in Table 2 along with corresponding linear velocities and accelerations at the center of mass of the head. The baseline head kinematics simulated here gives 90% probability of injury as per the acceleration injury thresholds developed by Rowson and Duma (2013) based on the data collected in instrumented football players.

**TABLE 2:** Head kinematics during pecking cycle.

Woodpecker pecking cycle	Time (ms)	Angular displacement at RP (rad/s)	Angular velocity at RP (rad/s)	Angular acceleration at RP (rad/s^2^)	Woodpecker		Human	
					Resultant velocity at CM of the head (m/s)	Resultant acceleration at the CM of the head (m/s^2^)	Resultant velocity at CM of the head (m/s)	Resultant acceleration at the CM of the head (m/s^2^)
Initial contact with wood (I)	0	0	0	0	0	0	0	0
Halfway rotation completed (II)	60	−0.32	−9.63	0	1.12	111	1.65	41.62
Extreme end reached and motion is reverted (III)	100	−0.58	0	408.43	0.20	2595	0.30	159.41
Before impact with tree (IV)	150	−0.18	14.45	0	1.66	253	2.44	68.29
Impact with tree (V)	160	0	0	−7056.73*	0.27	22468	0.67	4136.77*
Dwell (VI)	161–250	0	0	0	0**	0**	0**	0**

*The baseline head kinematics simulated here gives 90% probability of injury as per the acceleration injury thresholds developed by Rowson and Duma (2013) based on the data collected in instrumented football players. **Due to the stress wave propagation and inertial effects, the velocity and acceleration at the center of mass do not come to zero instantaneously but take ∼10 ms after an impact with the tree.

### Solution Scheme

The model was solved using the non-linear, transient, explicit dynamic scheme (Abaqus, Dassault Systemes Simulia Corp^®^). The total simulated time was 250 ms for a single cycle and 500 ms for 2 cycles. Note that each simulation included a dwell period, which ensured that the stress wave propagation effects within the brain were fully played out and captured. The time step used for explicit, dynamic simulations was on the order of 10^–7^ s, to ensure stability for each element ($\Delta t_{\text{stable}}=\frac{e\,l\,e\,m\,e\,n\,t\,l\,e\,n\,g\,t\,h}{d\,i\,l\,a\,t\,a\,t\,i\,o\,n\,a\,l\,w\,a\,v\,e\,s\,p\,e\,e\,d})$. The computational time required for woodpecker, human, and simplified woodpecker models were ∼4, ∼2, and ∼3 h of CPU time using 4 Intel Xeon Gold processors (processor speed 2.3 GHz, 4 GB memory per processor), for an integration time of 500 ms. Simulations were also performed to study the sensitivity of results to time integration scheme (implicit vs. explicit), element types (reduced vs. full integration), and viscous damping in dynamic simulation. The results are presented in the Supplementary Figures S2–S4.

### Statistical Analysis

For parametric studies, the model response has been evaluated using Pearson’s correlation coefficient (*r*), linear regression slope (*m*), and correlation score (CS). Details of these measures can be found in Ganpule et al. (2017) and Zhao and Ji (2019) and avoided here for brevity. Differences are considered to be statistically significant if one of the following conditions has been met.

r<0.90;  0.9<m<1.1;C⁢S<86

Note that *r*-values have been indicated in relevant figures and minimum values of *r*,*m*, *CS* have been specified throughout the text, wherever relevant.

## Results

### Model Validation

**FIGURE 4:**
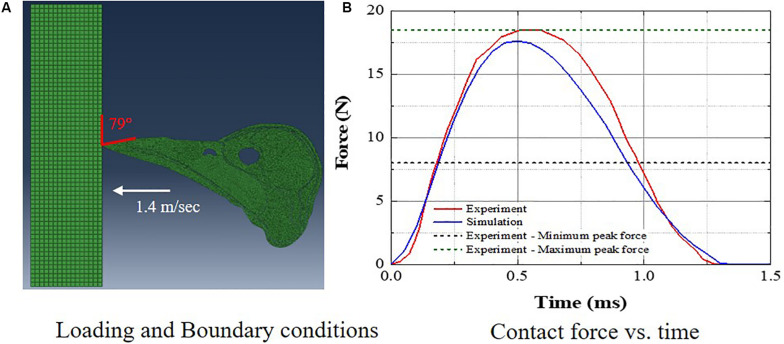
Comparison of woodpecker response against experimental data **(A)** loading and boundary conditions and **(B)** contact force vs. time.

The woodpecker and human head models were validated against relevant, available experimental data in the literature. Liu et al. (2017) recorded the pecking force during the pecking process in Great-spotted woodpecker. The woodpecker stood on the wooden board and pecked on it. The impact velocity at the time of impact was 1.4 m/s and the angle between the upper edge of the beak and the board was 79°. The simulations were performed with these initial conditions (Figure 4A) and a contact force was estimated (Figure 4B). Figure 4B shows the comparison of contact force between the simulation and experiment. The agreement between the simulation and experiment is reasonable. The peak force in the simulation lies between peak minimum and maximum force recorded in the experiment.

**FIGURE 5:**
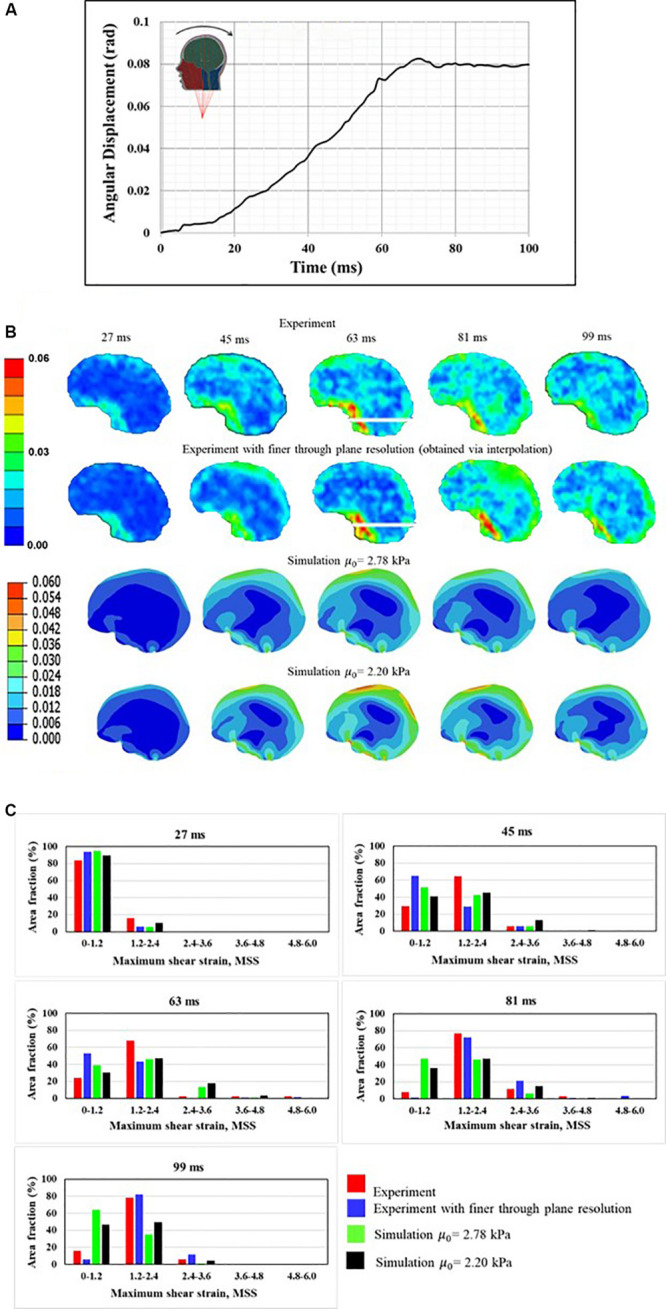
Comparison of human response against experimental data **(A)** boundary conditions, **(B)** qualitative comparison, and **(C)** quantitative comparison. Maximum shear strain, MSS measure is used for qualitative and quantitative companions.

Gomez et al. (2018) measured the full-field brain deformations in human volunteers during mild head rotation (peak head acceleration ∼300 rad/s^2^) using a custom made, MRI-compatible head motion apparatus. The apparatus fits inside the bore of an MRI machine and guides voluntary head motion to generate rotational motion in frontal-occipital direction. This results in a sagittal plane rotation about the neck (Figure 5A). To simulate the experiment, the measured angular displacement-time (Figure 5A) history was used as an input to the simulations. Figures 5B,C shows the comparison of response between a model and experiment. Both qualitative (Figure 5B) and quantitative (Figure 5C) agreement between a model and experiment is reasonable and commensurate with earlier investigations (Ji et al., 2015; Ganpule et al., 2017). The area fraction of strain in a given strain range is used as a quantitative measure, details can found in Ganpule et al. (2017) and are avoided here for brevity.

### Biomechanical Response During Pecking Cycle

**FIGURE 6:**
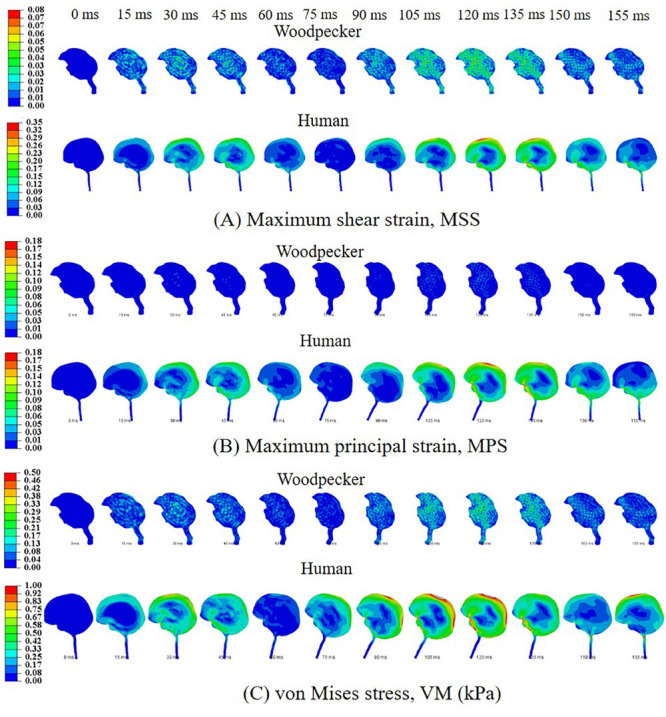
Biomechanical response of woodpecker and human brains corresponding to head rotation. **(A)** Maximum shear strain, MSS; **(B)** maximum principal strain, MPS; and **(C)** von Mises stress, VM (kPa).

**FIGURE 7:**
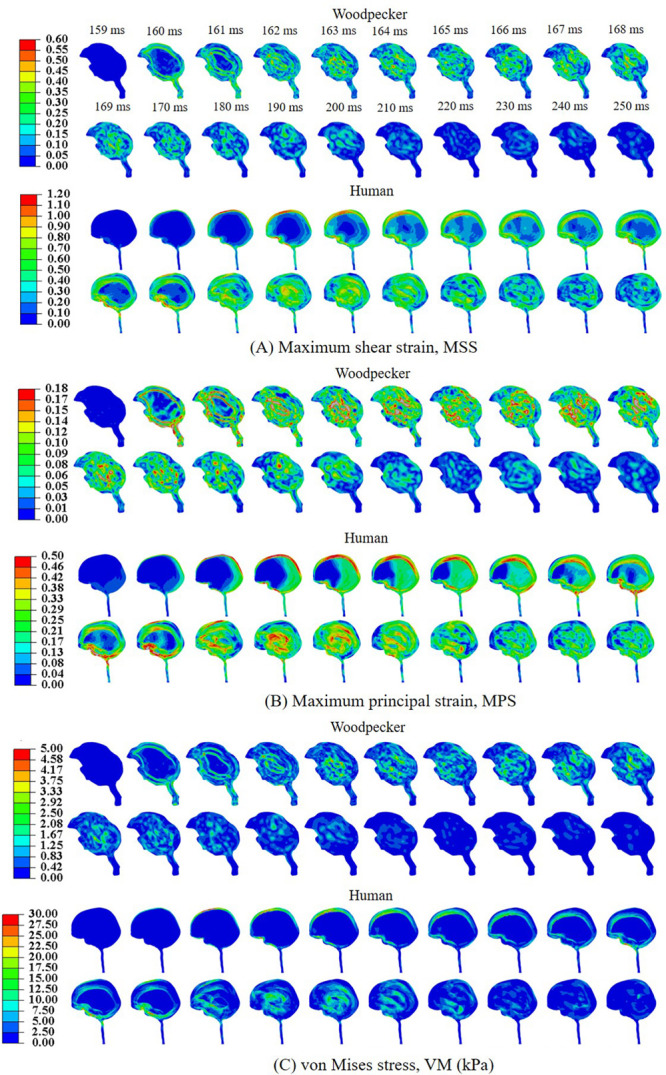
Biomechanical response of woodpecker and human brains corresponding to sudden deceleration and dwell. **(A)** Maximum shear strain, MSS; **(B)** maximum principal strain, MPS; and **(C)** von Mises stress, VM (kPa).

Biomechanical response during the pecking cycle is divided into two main phases: (i) head rotation and (ii) sudden deceleration and dwell. The results are presented separately for these two phases for ease of analysis and presentation. Figures 6A–C, respectively, show maximum shear strain (MSS), maximum principal strain (MPS) and von Mises stress (VM) in the woodpecker and human brains corresponding to head rotation. In the case of woodpecker, peak MSS, peak MPS, and peak VM are on the order of ±6%, ±3%, and 0.25 kPa, respectively. In the case of human, peak MSS, peak MPS, and peak VM stress are on the order of ±35%, ±18%, and 1 kPa, respectively. Figures 7A–C, respectively, show MSS, MPS, and VM in the woodpecker and human brains corresponding to sudden deceleration and dwell. In the case of woodpecker, peak MSS, peak MPS, and peak VM are on the order of ±60%, ±30%, and 5 kPa, respectively. In the case of human, peak MSS, peak MPS, and peak VM stress are on the order of ±120%, ±50%, and 30 kPa, respectively. The wave action continues to play out till ∼200 ms (i.e., ∼40 ms after the impact with the tree), as seen in the biomechanical response.

### Role of Geometric Features of the Woodpecker

**FIGURE 8:**
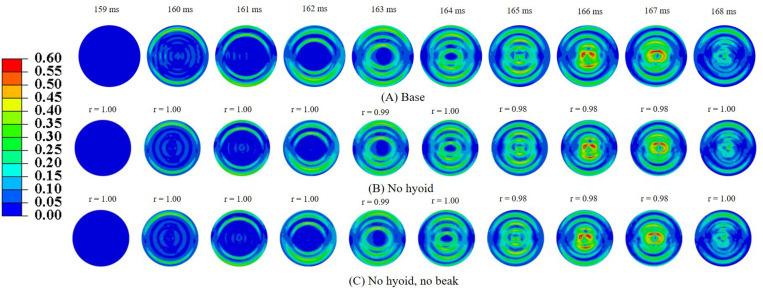
Maximum shear strain, MSS in the brain corresponding to sudden deceleration and dwell for a simplified woodpecker model **(A)** base, **(B)** no hyoid, and **(C)** no hyoid, no beak. Pearson’s correlation coefficient (*r*) values have been specified with respect to the base case.

In order to understand the role of geometric features of the woodpecker, simulations were performed using a simplified woodpecker model. The overall dimensions of the simplified woodpecker model were kept similar to the original woodpecker model. Simulations were performed for the following cases: (i) base model (ii) no hyoid, and (iii) no hyoid, no beak. Figure 8 shows MSS, corresponding to the sudden deceleration and dwell, in the brain of a simplified woodpecker for these three cases. Interestingly, results for the base model are qualitatively and quantitatively similar to the no hyoid (*r*_min_ = 0.98,*m*_min_ = 0.98,*m*_max_ = 1.00,*CS*_min_ = 99.17), no hyoid, no beak (*r*_min_ = 0.98,*m*_min_ = 0.98,*m*_max_ = 1.00,*CS*_min_ = 99.10) cases. This suggests that as compared to the other geometrical features, the size of the woodpecker plays a dominant role in governing the biomechanical response.

### Role of Pecking Frequency and Multiple Pecking Cycles

**FIGURE 9:**
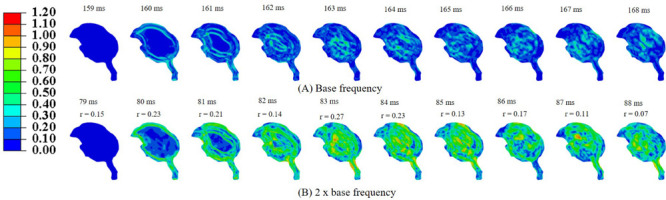
Effect of pecking frequency on biomechanical response **(A)** base frequency, **(B)** 2 × base frequency. Pearson’s correlation coefficient (*r*) values have been specified with respect to the base frequency.

**FIGURE 10:**
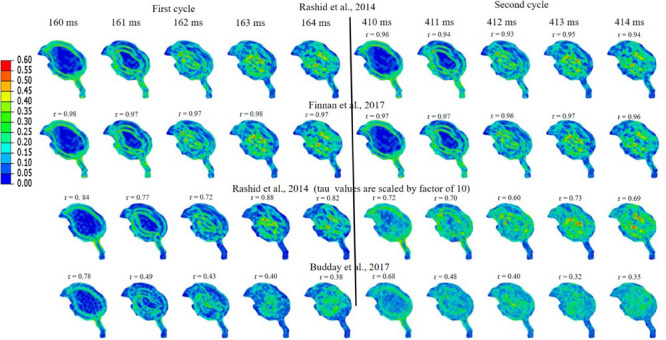
Effect of brain material properties and multiple cycles on biomechanical response. Sensitivity of maximum shear strain, MSS to material properties of the brain tissue is shown. For first cycle, Pearson’s correlation coefficient (*r*) values have been specified with respect to the Rashid et al. (2014). For second cycle, Pearson’s correlation coefficient (*r*) values have been specified with respect to the first cycle for each case.

Simulations were performed to study the effect of pecking frequency and multiple pecking cycles on biomechanical response. As the pecking frequency is doubled, the resulting deceleration is also doubled and hence the biomechanical response is much severe as indicated by the higher MSS values (Figure 9). The results are significantly different (*r*_min_ = 0.07,*m*_min_ = 0.14,*m*_max_ = 0.58,*CS*_min_ = 53.83). On the contrary, for the baseline material properties used in this work (Rashid et al., 2014), multiple pecking cycles do not significantly alter the biomechanical response as stress is significantly relaxed during the dwell period of ∼100 ms (Figure 10). To study the sensitivity of stress relaxation to material properties, additional simulations were performed (Figure 10) with various material properties of the brain tissue reported in the literature (Supplementary Table S1). The shear moduli values of Finan et al. (2017) and Budday et al. (2017) were scaled to match the baseline shear modulus of Rashid et al. (2014) at 5 ms (Supplementary Figure S5A), similar approach has been adopted by Zhao et al. (2018) that tend to produce good agreement with the experimental data. Note that, even though the shear modulus is scaled, there exists differences in hyperelastic response (Supplementary Figure S5B). As compared to Rashid et al. (2014), Finan et al. (2017) produces statistically similar (*r*_min_ = 0.97,*m*_min_ = 0.90,*m*_max_ = 1.00,*CS*_min_ = 96.05)response; whereas, Budday et al. (2017) produces a stiffer response (*r*_min_ = 0.38,*m*_min_ = 0.37,*m*_max_ = 0.66,*CS*_min_ = 81.49). For Rashid et al. (2014), when the decay constant τis increased by an order of magnitude, it produced a stiffer response (*r*_min_ = 0.72,*m*_min_ = 0.72,*m*_max_ = 0.87,*CS*_min_ = 91.07). As the decay constant τ is increased (i.e., slower relaxation), the peak strain values corresponding to second cycle is not significantly increased (% difference < 3%) with respect to first cycle (Figure 10 and Supplementary Table S2); however, the area fraction of strain having strains > 50% of peak values (ε_*MSS*_)*max* is increased by upto 9% (Figure 10 and Supplementary Figure S6). For second cycle, results are statistically similar with respect to the first cycle for Rashid et al. (2014) (*r*_min_ = 0.93,*m*_min_ = 0.93,*m*_max_ = 0.97,*CS*_min_ = 97.96)and Finan et al. (2017) (*r*_min_ = 0.96,*m*_min_ = 0.95,*m*_max_ = 0.97,*CS*_min_ = 98.63). Statistically significant differences in second cycle response are found for Budday et al. (2017) (*r*_min_ = 0.32,*m*_min_ = 0.28,*m*_max_ = 0.59,*CS*_min_ = 86.26)and Rashid et al. (2014) with τ = 10x*baseline* (*r*_min_ = 0.60,*m*_min_ = 0.66,*m*_max_ = 0.81,*CS*_min_ = 86.80).

### Relationship Between Woodpecker and Equivalent Human Response

**FIGURE 11:**
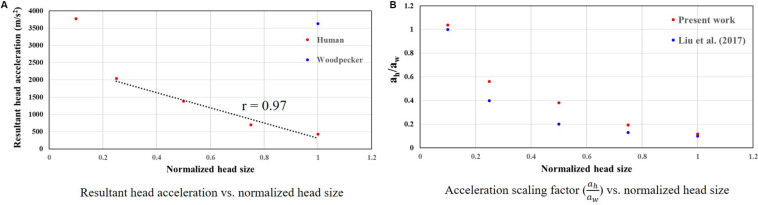
Relationship between woodpecker and equivalent human response. **(A)** Resultant head acceleration vs. normalized head size. **(B)** Acceleration scaling factor ($\frac{a_{\text{h}}}{a_{\text{w}}})$ vs. normalized head size. In case of human, head size is normalized with respect to the baseline (actual) human head size.

To develop a scaling relationship between human and woodpecker response, simulations were performed by scaling the head mass and head size. Both head mass and head size were scaled by the same amount, as the head mass scales linearly with head size (Bray et al., 1969; Rollins et al., 2010). Further, in these simulations, distance between the center of mass of the head and the reference point was kept same as that of the woodpecker. For each human head size, peak resultant brain acceleration was determined such that it produced similar peak stresses and strains as that of the woodpecker. While arriving at peak resultant brain acceleration, it was ensured that the stresses and strain in the human and woodpecker models were below injury threshold limits described earlier. Figure 11A shows the peak resultant acceleration at the center of mass of the brain as a function of human head size; head size is normalized with baseline (actual) human head size. Peak resultant acceleration at the center of mass of the brain for woodpecker is also shown. For the baseline (actual) sizes, the peak resultant brain accelerations are 429 and 3,630 m/s^2^ for human and woodpecker, respectively. As the human head size is reduced, tolerable resultant brain acceleration is increased. The response is linear (*R* = 0.97) until the normalized head size of 0.25, as the head size decreases further response becomes non-linear. Interestingly, for the normalized human head size of 0.1 (that produces approximately same mass and size as that of woodpecker), tolerable acceleration in human is comparable to tolerable acceleration in woodpecker.

**FIGURE 12:**
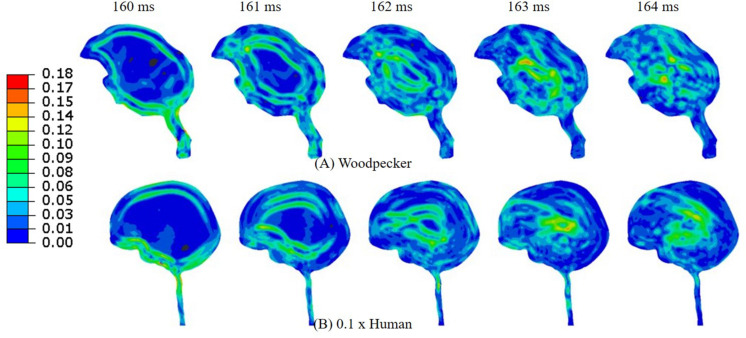
Comparison of **(A)** woodpecker and **(B)** human response when the human head mass and head size was scaled by a factor of 0.1.

Figure 11B shows the acceleration scaling factor ($\frac{a_{\text{h}}}{a_{\text{w}}})$ as a function of the human head size that gives an equivalent response as that of the woodpecker. Corresponding data from Liu et al. (2017) is also plotted. For the baseline (actual) head size, the acceleration scaling factor is 0.11. The scaling factor increases as the head size is reduced. The value of the scaling factor is 1.03 for normalized head size of 0.1. As expected, for this head size, biomechanical response between a human and a woodpecker are similar (Figure 12).

## Discussion

### Comparison of Biomechanical Response Between Woodpecker and Human

In this work, one-to-one comparison of biomechanical response between a woodpecker and human has been performed using 2D finite element head models. 2D models have been used that are easy to build and computationally efficient as compared to the 3D models. 2D models used in this work contain all geometric features that are shown to be critical for biomechanical analysis (Wang et al., 2011; Zhu et al., 2012; Liu et al., 2015b, 2017; Ganpule et al., 2017). 2D model responses have been compared against experimental data for woodpecker and human, quality of agreement between the model and the experiment is reasonable (Figures 4, 5).

We simulated a full pecking cycle that consists of two important phases (i) head rotation and (ii) sudden deceleration and dwell. Our results indicate that corresponding to these phases the strains and stresses in the brain of a woodpecker are smaller by a factor of up to six as compared to a human brain (Figures 6, 7). The biomechanical response obtained in this work is commensurate with the biomechanical response obtained using 3D models (Liu et al., 2015b; Ganpule et al., 2017). Further, the stress distribution in the beak, hyoid, and skull of a woodpecker (Supplementary Figure S7) is comparable to the stress distribution in 3D models (Zhu et al., 2012; Liu et al., 2015b).

In order to gain insights into the source of strain and stress reduction in a woodpecker, additional simulations were performed with a simplified woodpecker model and key geometrical features were omitted (Figure 8). Our results indicate that the strain and stress reduction in the brains of a woodpecker is mainly attributed to its smaller size (Figure 8). Results were statistically similar (*r*_min_ = 0.98,*m*_min_ = 0.98,*m*_max_ = 1.00,*CS*_min_ = 99.10) for baseline, no hyoid, and no hyoid, no beak cases. When the human head model was scaled to an approximate size of woodpecker, the responses between human and woodpecker brains were similar in terms of resultant head acceleration (Figure 11), and strains (Figure 12). This reinforces that the head size plays a vital role in brain biomechanics. Our results are consistent with some of the findings in the literature. Gibson (2006) has derived a relationship between the acceleration of the head, contact area of the brain with the skull, and stress in the brain. Based on the analysis, stress reduction in the woodpecker brain has been attributed to its smaller size. Gibson’s (2006) approach is oversimplified due to the gross assumptions regarding contact of the brain within the skull. Further, their analysis is based on the head kinematics alone without taking into account stress wave propagation effects within the head that are critical during impact loading (Ramesh, 2008). Kleiven and von Holst (2002), using the 3D human head model, have found that for the same input acceleration, the stress in the brain has reduced as the head size is decreased. Margulies et al. (1990) subjected the human and baboon skulls filled with the surrogate brain material to the rotational loading and measured the strains in the surrogate brain material. They found lower strains in the surrogate brain in the case of the baboon as compared to the human.

In our simulations, the omission of key geometric features (i.e., beak and hyoid bone) in a simplified woodpecker model (Figure 8) did not change the biomechanical response significantly. Liu et al. (2015b) performed finite element based biomechanical analysis on 3D woodpecker head. They found a marginal reduction in shear stress and strain energy in the brain of a woodpecker with the presence of hyoid bone. Other investigations (Wang et al., 2011; Zhu et al., 2012) though hypothesize the role of beak and hyoid bone during pecking, do not explicitly demonstrate the influence of these structures or lack of them on the brain response.

### Implications for Injury

Several injury criteria for traumatic brain injury, especially diffuse axonal injury (DAI), have been proposed in the literature. Wright et al. (2013), based on the work of Bain and Meaney (2000), identified the threshold value of 18% axonal tensile strain as an onset of DAI. Giordano and Kleiven (2014), based on finite element accidental reconstructions from the American National Football League (NFL), defined the threshold value of 13% axonal tensile strain as an onset of DAI. Zhang et al. (2004), based on finite element reconstructions of 24 head-to-head collisions from NFL, proposed a shear stress value of 7.8 kPa as the tolerance level for a 50% probability of sustaining a mild TBI. Studies focusing on scaling relationships (Gibson, 2006; Liu et al., 2017) assume that injury occurs at the same stress (and strain) levels for the woodpecker and human, and accelerations are scaled. Thus, we hypothesize that the injury will occur at the same stress (and strain) levels for the woodpecker and human.

Based on axonal strain based criterion, the human brain exceeds the injury threshold limit during both the head rotation (Figure 6B) and sudden deceleration-dwell phases (Figure 7B); whereas using shear stress based criterion, the human brain exceeds the injury threshold limit during the sudden deceleration-dwell phase (Figure 7C). On the other hand, based on axonal strain based criterion, woodpecker exceeds the injury threshold limit during the sudden deceleration-dwell phase (Figure 7B). Using shear stress based criterion, the woodpecker brain does not exceed the injury threshold limit during the entire pecking cycle (Figures 6C, 7C).

A comparison of the biomechanical response of woodpecker against axonal strain injury based thresholds suggests that even woodpecker exceeds existing brain injury thresholds during the pecking cycle. Recently, Farah et al. (2018) have found the accumulation of tau-protein, neuropathology implicated in human CTE (McKee et al., 2016), in the heads of woodpeckers as compared to the controls. Thus, the biomimicry attempts of the woodpecker should be viewed with caution and should be supported with detailed analysis.

### Effect of Pecking Frequency and Pecking Cycle

Woodpeckers do peck at different pecking frequencies. As the pecking frequency is increased, the resulting biomechanical response is proportionally severe in the form of higher strain and stress within the brain (Figure 9). These differences were statistically significant (*r*_min_ = 0.07,*m*_min_ = 0.14,*m*_max_ = 0.58,*CS*_min_ = 53.83). The biomechanical response during multiple pecking cycle is dependent on the choice of relaxation time constants (Figure 10). Interestingly, as relaxation times are increased, peak strain magnitude is not significantly changed (% difference < 3%) with respect to the first cycle (Figure 10 and Supplementary Table S2). However, the area fraction of strain having strains > 50% of peak values is increased by upto 9% (Figure 10 and Supplementary Figure S6), suggesting damage potential over a larger area. Further, statistically significant differences (*r*_min_ = 0.38,*m*_min_ = 0.37,*m*_max_ = 0.66,*CS*_min_ = 81.49)in the response were seen when decay constant τ was scaled by factor of 10 or higher. The material properties of the brain tissue used in this work are based on characterization in human and porcine brains. Our results suggest that characterizing material response in the woodpecker brain will be critical to faithfully simulate the woodpecker biomechanical response. For the relatively faster relaxation on the order of ∼50 ms, the dwell period is beneficial in terms of relaxing the stresses before woodpecker begins the next pecking cycle.

### Scaling Relationship Between Woodpecker and Human Response

Scaling relationship between human and woodpecker response was studied by scaling the head mass and head size. Resultant acceleration at the center of mass of the brain was determined such that it produced similar peak stresses and strains as that of the woodpecker. Further, the stresses and strains were below the existing injury threshold limits. For the baseline (actual) sizes, peak resultant brain accelerations of 429 and 3,630 m/s^2^ in human and woodpecker, respectively, produce a similar biomechanical response (Figure 11A). Corresponding linear velocities at the center of mass of the head are 0.2 and 1 m/s in human and woodpecker, respectively. This is consistent with finding of Liu et al. (2015b), who reported an acceleration threshold value of ∼4,120 m/s^2^ in the woodpecker for an impact velocity of 1 m/s. As the human head size is reduced, accelerations required to produce similar stresses as that of woodpecker are increased. We obtain an acceleration scaling factor ($\frac{a_{\text{h}}}{a_{\text{w}}})$of 0.11 for baseline human head size and scaling factor of 1.03 when the human head size approaches the woodpecker head size (Figure 11B). The values of the scaling factor at extreme ends (i.e., normalized head sizes 1, 0.1) are consistent with values reported by Liu et al. (2017). For the intermediate sizes, the absolute values of the scaling factor deviate from Liu et al. (2017), however, trend remains similar. While arriving at an equation for acceleration scaling between a woodpecker and human, Liu et al. (2017) assumed perfectly hemispherical geometries for human and woodpecker. Gibson (2006) suggests that the scaling factor can change based on the orientation of the brain within the skull, actual contact area between the skull and the brain, and mass of the head.

### Limitations

This work has several limitations. Numerous studies report that the beak and hyoid of a woodpecker consist of a hierarchical structure with a unique structure-property relationship. In this work, we do not explicitly consider a hierarchical structure of a beak and hyoid. We, however, note that the mechanical properties of hierarchical structures of beak and hyoid are not drastically different (within the same order of magnitude). Hence, we do not expect a significant difference in biomechanical response with explicit modeling of hierarchical structures of beak and hyoid. Further, a 2D model of woodpecker does not consider the asymmetric design of the beak, an evolutionary trait.

## Data Availability Statement

The datasets generated for this study are available on request to the corresponding author.

## Author Contributions

SG conceptualized the manuscript and developed the initial 2D model of a woodpecker and human heads. SS and KS improved on the initial 2D head models. SS, SG, and KS performed the simulations and generated the data. SG and SS analyzed the results. SG wrote the manuscript with assistance from SS. All authors contributed to the article and approved the submitted version.

## Conflict of Interest

The authors declare that the research was conducted in the absence of any commercial or financial relationships that could be construed as a potential conflict of interest.

## Supplementary Material

FIGURE S1 Sensitivity of results to mesh size **(A)** woodpecker and **(B)** human. Maximum shear strain, MSS is shown. A similar agreement is seen for Maximum principal strain, MPS, and von Mises stress, VM.

FIGURE S2 Sensitivity of woodpecker results to time integration scheme and element types. For first cycle, Pearson’s correlation coefficient (*r*) values have been specified with respect to the explicit, reduced integration scheme. For second cycle, Pearson’s correlation coefficient (*r*) values have been specified with respect to the first cycle for each case.

FIGURE S3 Sensitivity of woodpecker results to time integration scheme in a coarser, simplified model of a woodpecker. The same time step is used for implicit and explicit simulations. For first cycle, Pearson’s correlation coefficient (*r*) values have been specified with respect to the explicit, reduced integration scheme. For second cycle, Pearson’s correlation coefficient (*r*) values have been specified with respect to the first cycle for each case.

FIGURE S4 Sensitivity of woodpecker results to viscous damping. For first cycle, Pearson’s correlation coefficient (*r*) values have been specified with respect to the base case. For second cycle, Pearson’s correlation coefficient (*r*) values have been specified with respect to the first cycle for each case.

FIGURE S5 **(A)** Viscoelastic response of brain tissue. **(B)** Hyperelastic response of brain tissue for various material properties reported in the literature.

FIGURE S6 Effect of multiple cycles on biomechanical response. Quantitative comparison of the sensitivity of simulation results to the material properties of brain tissue.

FIGURE S7 Contour plot depicting von Mises stress, VM (MPa) in the woodpecker head. Response is comparable to the studies of Zhu et al. (2012) and Liu et al. (2015b).

TABLE S1 Material properties of the brain tissue used for parametric study.

TABLE S2 Sensitivity of stress relaxation to material properties.
