# Supplementary figures and images for: Biomechanical Analysis of Woodpecker Response During Pecking Using a Two-Dimensional Computational Model
Source: Front Bioeng Biotechnol. 2020 Jul 17;8:810. doi: 10.3389/fbioe.2020.00810 (PMC7379169; doi:10.3389/fbioe.2020.00810)

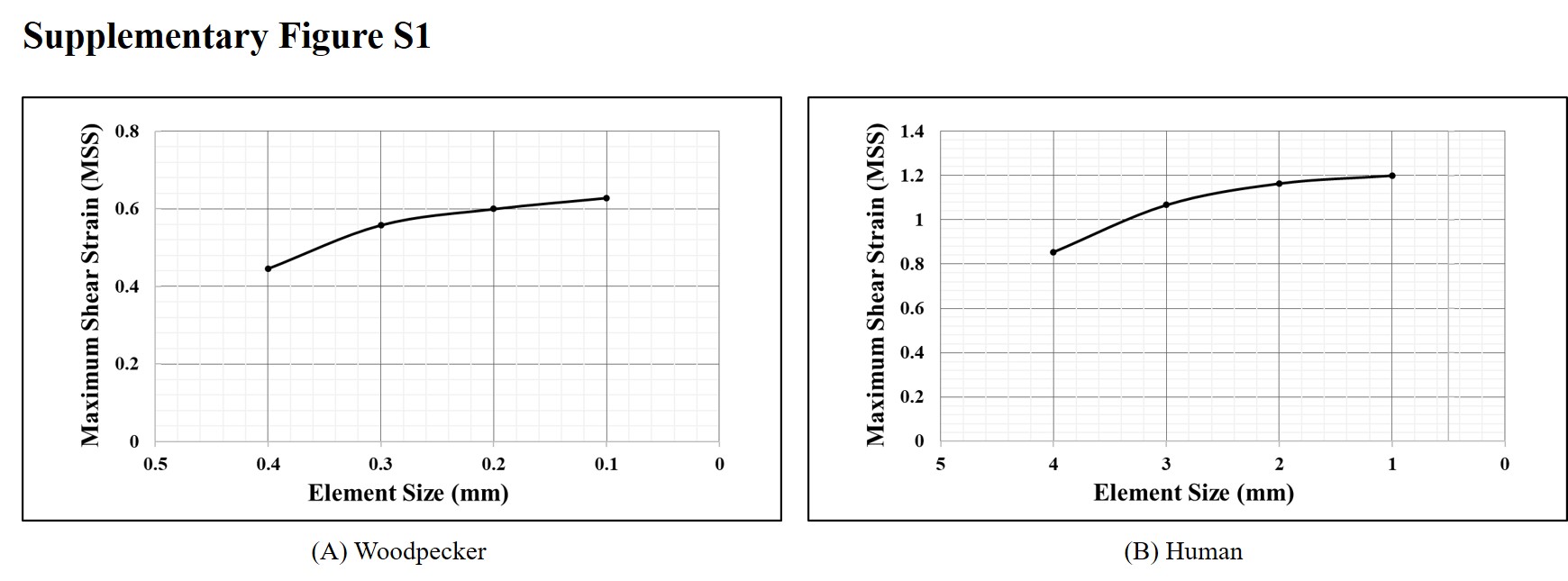

Supplement: FIGURE S1 — Sensitivity of results to mesh size (A) woodpecker and (B) human. Maximum shear strain, MSS is shown. A similar agreement is seen for Maximum principal strain, MPS, and von Mises stress, VM. [file Image_1.JPEG]

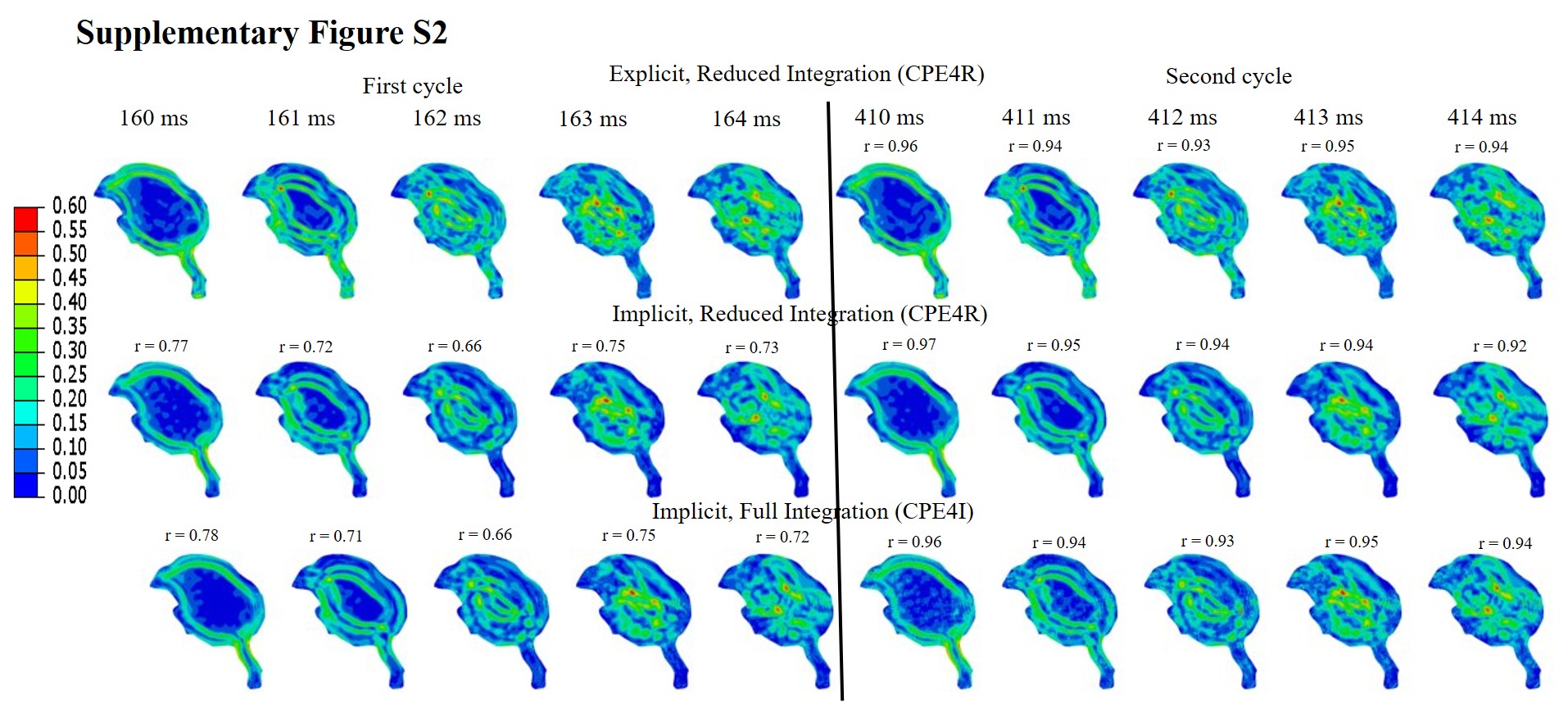

Supplement: FIGURE S2 — Sensitivity of woodpecker results to time integration scheme and element types. For first cycle, Pearson’s correlation coefficient (r) values have been specified with respect to the explicit, reduced integration scheme. For second cycle, Pearson’s correlation coefficient (r) values have been specified with respect to the first cycle for each case. [file Image_2.JPEG]

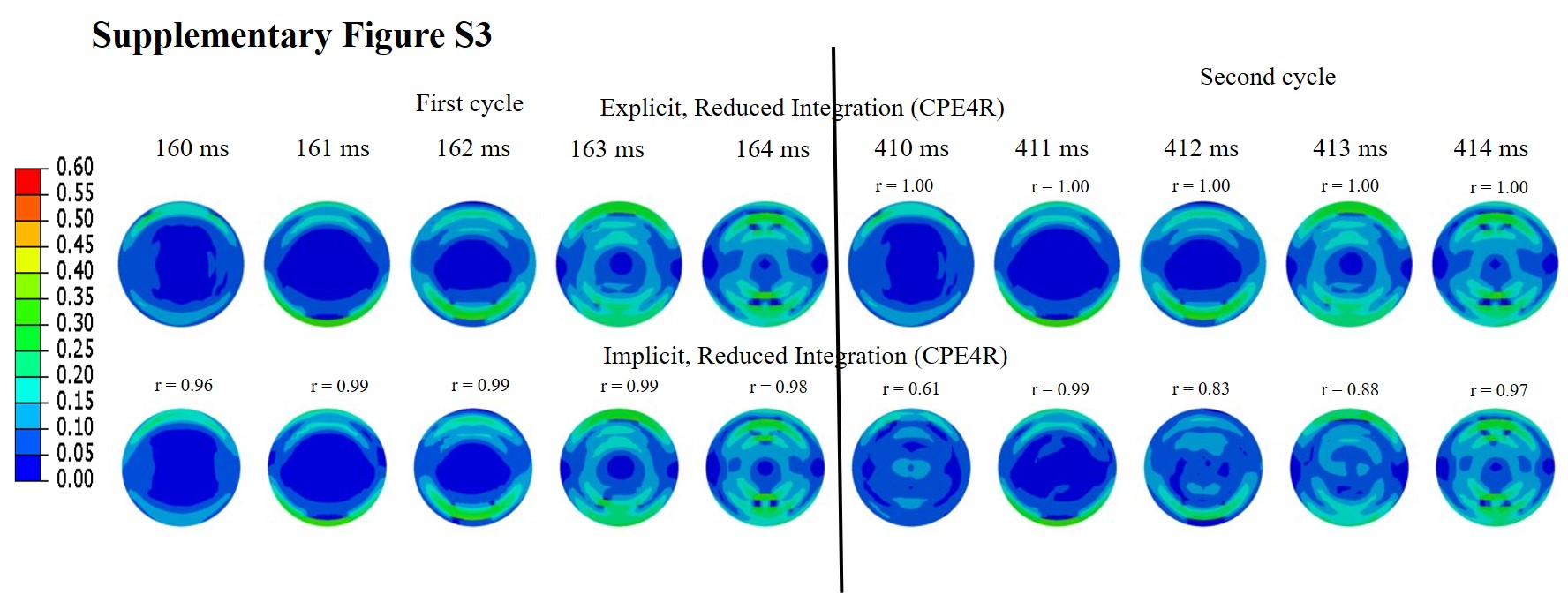

Supplement: FIGURE S3 — Sensitivity of woodpecker results to time integration scheme in a coarser, simplified model of a woodpecker. The same time step is used for implicit and explicit simulations. For first cycle, Pearson’s correlation coefficient (r) values have been specified with respect to the explicit, reduced integration scheme. For second cycle, Pearson’s correlation coefficient (r) values have been specified with respect to the first cycle for each case. [file Image_3.JPEG]

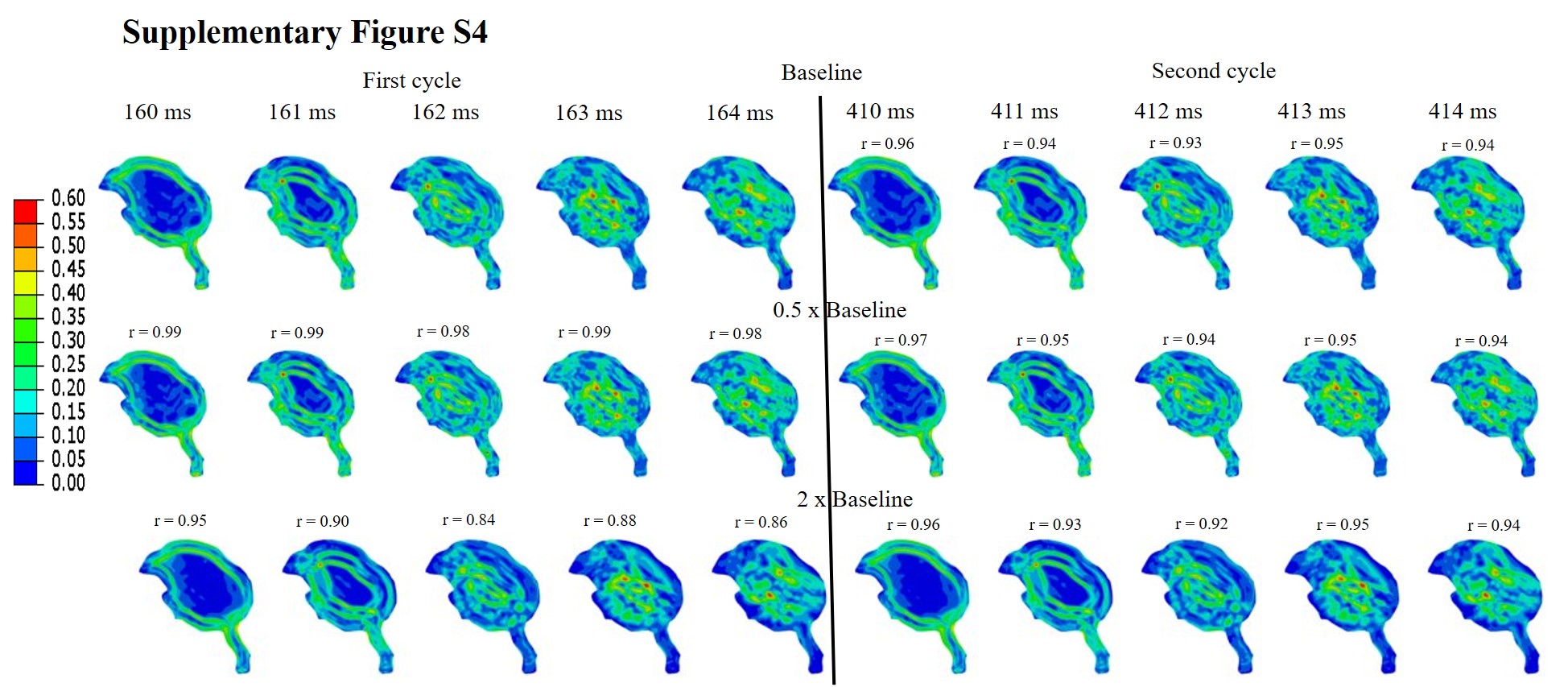

Supplement: FIGURE S4 — Sensitivity of woodpecker results to viscous damping. For first cycle, Pearson’s correlation coefficient (r) values have been specified with respect to the base case. For second cycle, Pearson’s correlation coefficient (r) values have been specified with respect to the first cycle for each case. [file Image_4.JPEG]

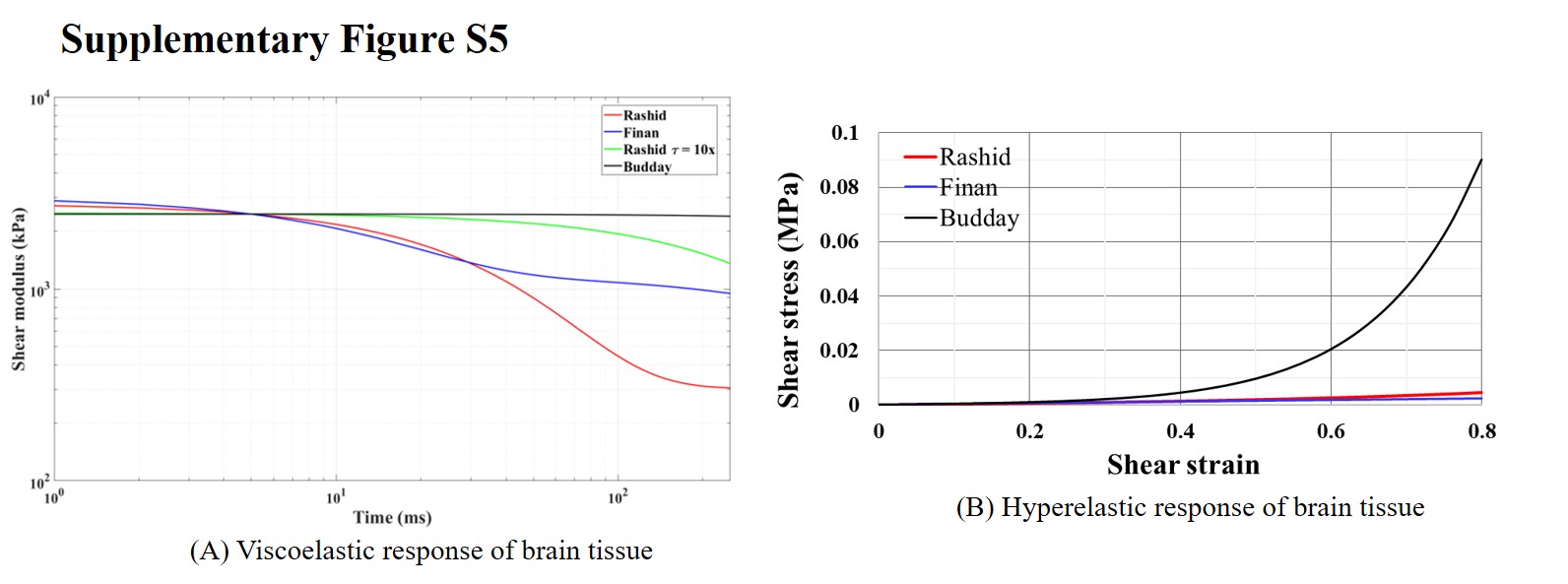

Supplement: FIGURE S5 — (A) Viscoelastic response of brain tissue. (B) Hyperelastic response of brain tissue for various material properties reported in the literature. [file Image_5.JPEG]

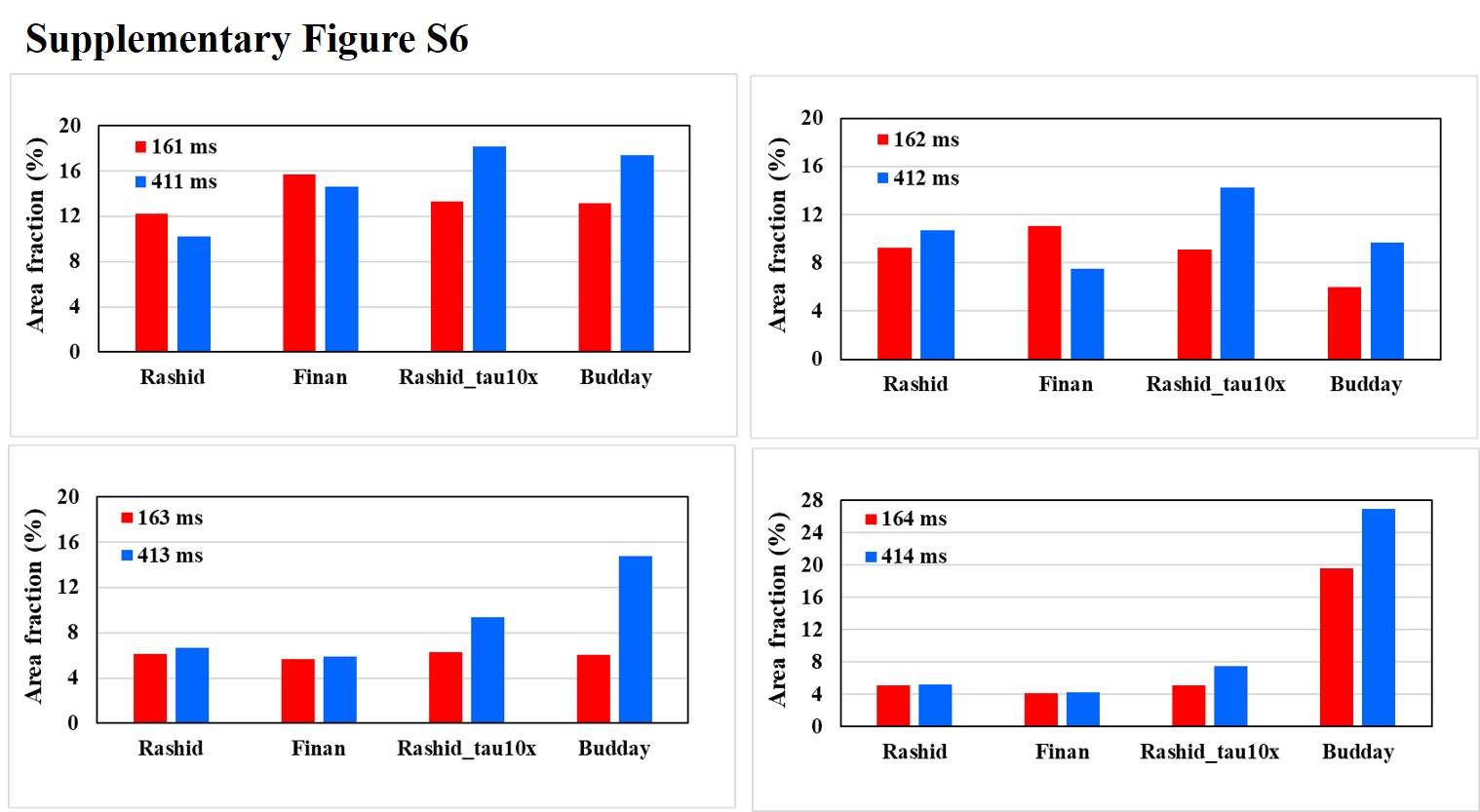

Supplement: FIGURE S6 — Effect of multiple cycles on biomechanical response. Quantitative comparison of the sensitivity of simulation results to the material properties of brain tissue. [file Image_6.JPEG]

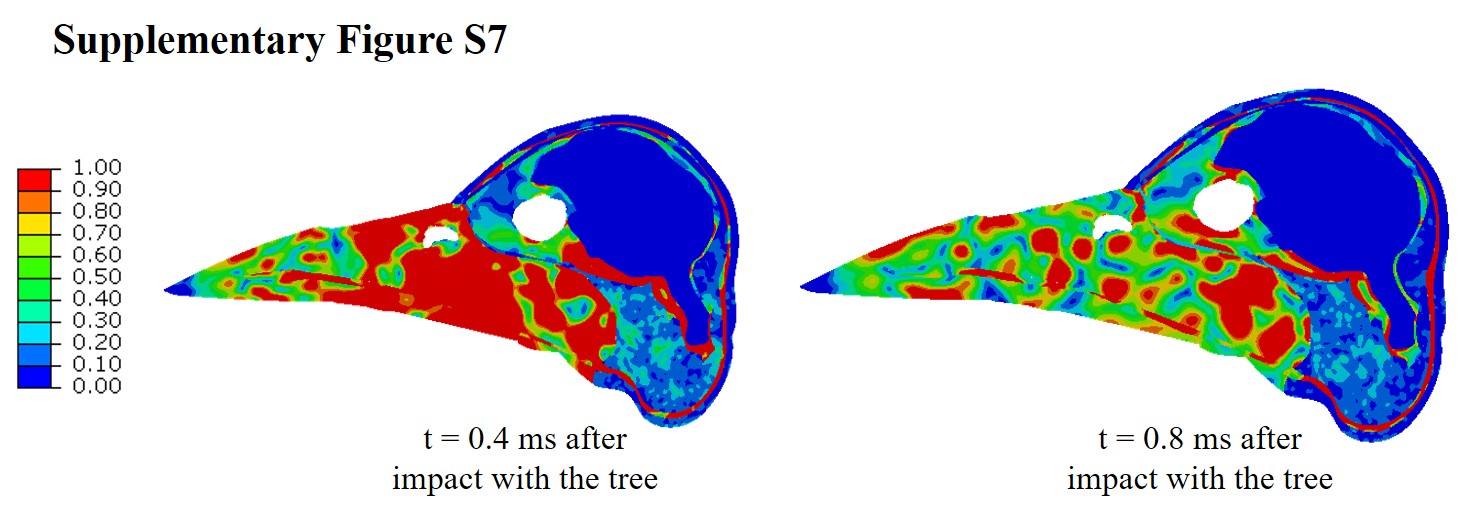

Supplement: FIGURE S7 — Contour plot depicting von Mises stress, VM (MPa) in the woodpecker head. Response is comparable to the studies of Zhu et al. (2012) and Liu et al. (2015b). [file Image_7.JPEG]
