# Supplementary material for: Biomechanical Analysis of Woodpecker Response During Pecking Using a Two-Dimensional Computational Model
Source: Front Bioeng Biotechnol. 2020 Jul 17;8:810. doi: 10.3389/fbioe.2020.00810 (PMC7379169; doi:10.3389/fbioe.2020.00810)
Supplement: TABLE S1 — Material properties of the brain tissue used for parametric study. [file Data_Sheet_1.pdf]

Supplementary Material

Sensitivity of woodpecker results to time integration scheme and element types:

Supplementary Figure S2 shows the sensitivity of woodpecker simulation results to the time integration scheme (implicit vs. explicit). As compared to the explicit time integration scheme, results are smeared out using the implicit time integration scheme. This is due to the fact that a default time step used in the implicit simulation is ~1000 times larger than the minimum time step required for the explicit simulation. Hence, in the implicit simulation, peak values (MSS, MPS, VM) are underpredicted by ~25%. Further, difference between implicit and explicit time integration schemes was statistically significant ($r_{min} = 0.66, m_{min} = 0.52, m_{max} = 0.68, CS_{min} = 87.75$). Further, differences between implicit and explicit time In the figure, results for MSS are only shown for brevity. For woodpecker model, the time required to run the implicit simulation using the same time step as that of the explicit simulation is notoriously high (estimated time ~765 CPU hours). To fully address this issue, we performed additional simulations using coarser, simplified model of a woodpecker (574 elements) with same time step ($\Delta t = 7.842 \times 10^{-7}$) in both implicit and explicit simulations (Supplementary Figure S3), and it produced statistically similar response ($r_{min} = 0.96, m_{min} = 0.96, m_{max} = 1.03, CS_{min} = 98.66$). The simplified model required 23 hours of CPU time.

Reduced (CPE4R) and full integration (CPE4I) implicit simulations produced statistically similar response ($r_{min} = 0.98, m_{min} = 0.96, m_{max} = 0.99, CS_{min} = 99.12$). Note that, Abaqus (Abaqus, Dassault Systemes Simulia Corp®) explicit solver with plane stain condition only has reduced integration elements in the element library.

Sensitivity of woodpecker results to viscous damping:

Supplementary Figure S4 shows the sensitivity of woodpecker simulation results to viscous damping. The damping is scaled by factors of 0.5 and 2 from the baseline. For scale factor 0.5,

results are statistically similar ($r_{min} = 0.98, m_{min} = 0.95, m_{max} = 0.99, CS_{min} = 98.37$). For the scale factor of 2, peak values are reduced by ~25% and difference in the response is statistically significant ($r_{min} = 0.84, m_{min} = 0.75, m_{max} = 0.90, CS_{min} = 93.63$)

Sensitivity of woodpecker simulation results to material properties:

For a detailed description, see section 3.4.

Supplementary Table ST1: Material properties of the brain tissue used for parametric study

**Baseline:** Rashid et al. 2014 (Rashid et al., 2014)	$\rho = 1040$ kg/m^3<br>$\mu_0 = 2780$ Pa, $\mu_\infty = 303.3\ Pa$,<br>$\alpha = 6.0$, $g_1 = 0.5663$, $g_2 = 0.3246$, $\tau_1 = 0.0350\ s$, $\tau_2 = 0.0351$ s
Finan et al. 2017 (Finan et al., 2017)	$\rho = 1040$ kg/m^3<br>$\mu_0 = 1062.1$ Pa, $\mu_\infty = 99.1\ Pa$,<br>$g_1 = 0.603$, $g_2 = 0.169$, $g_3 = 0.136$,<br>$\tau_1 = 0.014\ s$, $\tau_2 = 0.425\ s$,<br>$\tau_3 = 5.290\ s$
Budday et al. 2017 (Budday et al., 2017)	$\rho = 1040$ kg/m^3<br>$\mu_0 = 2960$ Pa, $\mu_\infty = 360\ Pa$,<br>$\alpha = -16.07$, $g_1 = 0.601$, $g_2 = 0.277$,<br>$\tau_1 = 5.719$, $\tau_2 = 851.000$

Supplementary Table ST2: Sensitivity of stress relaxation to material properties

	Peak maximum shear strain (MSS)												
	First cycle						Second cycle						
	160 ms	161 ms	162 ms	163 ms	164 ms	**Maximum 160-164**	410 ms	411 ms	412 ms	413 ms	414 ms	**Maximum 410-414**	**% change corresponding to first and second cycle***
**Rashid**	0.55	0.46	0.48	0.61	0.59	**0.61**	0.57	0.51	0.49	0.62	0.60	**0.62**	0.45
**Finan**	0.63	0.42	0.47	0.63	0.61	**0.63**	0.63	0.43	0.51	0.64	0.62	**0.64**	0.80
**Rashid_tau10x**	0.54	0.45	0.50	0.63	0.60	**0.63**	0.59	0.52	0.55	0.64	0.65	**0.65**	2.58
**Budday**	0.41	0.42	0.51	0.51	0.45	**0.51**	0.41	0.40	0.51	0.44	0.40	**0.51**	0.38

* % change corresponding to the first and second cycle is calculated based on the difference between absolute peak value seen during 160-164 ms and absolute peak value seen during 410-414 ms.
